# Effect of Chemotherapy Treatment on Overall Survival of Colon Cancer Patients Using Propensity Score Matching

**DOI:** 10.7759/cureus.83892

**Published:** 2025-05-11

**Authors:** Corey Hebert, Lawerence Shi, Runhua Shi

**Affiliations:** 1 Medicine, Louisiana State University Health Sciences Shreveport, Feist-Weiller Cancer Center, Shreveport, USA; 2 Medicine, Louisiana State University Health Sciences Center, New Orleans, USA; 3 Clinical Informatics, Louisiana State University Health Sciences Shreveport, Feist-Weiller Cancer Center, Shreveport, USA

**Keywords:** chemotherapy, colon cancer, epidemiology, overall survival, propensity score matching

## Abstract

Background

Colon cancer is a common cause of cancer and cancer-associated deaths in the United States. Many factors can influence the overall survival (OS) of patients with colon cancer, such as patient demographics, clinical presentation, and treatment characteristics. The goal of this study was to assess the influence of chemotherapy on OS in patients with colon cancer with similar baseline characteristics.

Materials and methods

A total of 70,876 patients with stage II and III colon cancer, confirmed by pathology, over the age of 18, and who were diagnosed with colon cancer between 2004 and 2019, were selected from the de-identified National Cancer Database (NCDB). All of the patients included in the study underwent surgical treatment. Patients who received hormonal therapy, radiation therapy, immunotherapy, palliative care, or any treatment modality besides chemotherapy were excluded from the analysis. Calculation of the propensity score was performed by computing the probability of patients being in the chemotherapy group using logistic regression. The propensity score matching (PSM) was done via the PSMATCH procedure with the SAS software (SAS Institute, Cary, NC, USA) on patients who received chemotherapy compared to patients who received a treatment other than chemotherapy. The greedy nearest neighbor matching method was then utilized to match one chemotherapy patient to one non-chemotherapy patient with a caliper of 0.2. An exact match was done for sex, race, tumor stage, and year diagnosed at the time of patient diagnosis. Multivariate Cox regression analysis was then used to estimate the effect of chemotherapy on OS before and after PSM.

Results

A total of 70,876 patients were included in the study before PSM, with 44,992 receiving chemotherapy and 25,884 not receiving chemotherapy. Before PSM, the OS was 17.55 years for patients who received chemotherapy, compared to 14.12 years for those who did not. After matching 23,356 patients, the OS was 17.77 years for patients who received chemotherapy and 12.18 years for those who did not. Following PSM, patients who received chemotherapy were 46% less likely to die compared to those who did not receive chemotherapy.

Conclusion

Our findings, per the PSM method, demonstrate that receiving chemotherapy can be a significant predictor of OS among patients with stage II and III colon cancer. Other variables such as tumor stage, age, and insurance type were also found to be significant predictors of OS in colon cancer patients. Prospective clinical studies are necessary and should be performed to determine the true effects of chemotherapy on OS.

## Introduction

Colorectal cancer is the fourth most common cancer in the United States, with an incidence of 36.5 per 100,000 men and women per year between 2017 and 2021 [1]. The American Cancer Society predicts that there will be an estimated 106,590 new cases and 53,010 deaths expected in 2024 alone [2]. Colon cancer ranks third in cancer-related deaths, with a death rate of 12.9 deaths per 100,000 men and women between 2018 and 2022 [1]. Per 100,000, Blacks had the highest number of new cases and deaths among both genders [2]. For overall cases of colorectal cancer, the National Cancer Institute Surveillance, Epidemiology, and End Results (SEER) data from 2014 to 2020 reported a 65.0% five-year survival rate [1]. The SEER reported the highest percentage of new cases and deaths among people aged 65 to 74, with a median age of 66 at diagnosis and 72 at death. Since the 1980s, the incidence of colorectal cancer has steadily decreased, and survivorship has increased in older adults, likely due to improvements in lifestyle-related risk factors and better screening guidelines [2]. Because of these improvements, there are currently over 1.5 million colon cancer survivors [2]. It is important to note that among individuals younger than 55 years, the incidence of colon cancer has been steadily increasing by 1% to 2% annually since the mid-1990s [2]. 

Several factors may influence the overall survival (OS) of colon cancer patients, including patient demographics, clinical presentation, and treatment characteristics, all of which have been explored in previous research [2]. Age at diagnosis is a significant predictor of OS, as increased patient age leads to decreased OS [3, 4]. Females are associated with better survival than males [4], even among patients with metastatic disease [5]. Blacks are often diagnosed at later stages and experience disparities in screening, leading to worse OS when compared to their White counterparts [6-9]. Patients presenting with comorbidities at the time of diagnosis have a worse OS [7]. 

Other studies have shown that, at diagnosis, a higher stage was associated with lower survival [8] and that an earlier stage is linked to better survival when treatment is not delayed [4,9]. Higher tumor grade demonstrates adverse histological characteristics and is a poor prognostic factor in colon cancer [9]. Diagnoses of colon cancer in recent years are linked to a higher OS when compared to earlier diagnoses, likely due to newer and advanced treatments [4].

Centralization of cancer treatment has increased travel distance for patients to receive healthcare [10] and can be predictive, as patients with longer commutes are often diagnosed with colon cancer at later stages with worse OS, likely because distance serves as a barrier to early screening or preventative treatment [11]. Colon cancer patients from metropolitan areas have better OS compared to those from urban or rural areas, and patients from non-urbanized areas are also more likely to refuse treatment [12]. Academic medical centers are associated with better OS for patients [13] compared to community healthcare centers, likely due to better treatment resources and better use of surgery and chemotherapy [14]. Insurance status is an important predictor of OS, as Medicaid patients who may receive the same treatment as their privately insured counterparts have worse OS, as cancer is often caught at a later stage [6,15,16]. Colon cancer patients with low income or from poor socioeconomic conditions had a higher overall risk of death when compared to those with higher economic status [17,18]. 

The aim of this study is to assess the effect that chemotherapy plays on OS in colon cancer patients with comparable baseline demographic and clinical characteristics. To attain comparable baseline characteristics, this study used the propensity score matching (PSM) method to ensure even baseline characteristics between chemotherapy groups [19,20]. 

## Materials and methods

This retrospective study included 78,843 patients with stage II or III colon cancer who were registered in the de-identified National Cancer Database (NCDB) between 2004 and 2020 and with follow-up until December 31, 2020. The NCDB file records approximately 70% of new cancer diagnoses at the institutional level in the United States. The codes associated with a diagnosis of colon cancer in association with the International Classification of Disease for Oncology, third edition (ICD-O-3), i.e., the ICD-O-3 code: primary site recodes C180-189 verified by pathology (ICD-O-3 code: histological type recodes 8000, 8010, 8012-8014, 8032-8033, 8041, 8050, 8070-8072, 8075, 8083, 8140-8141, 8143-8144, 8210-8211, 8213, 8220-8211, 8220-8221, 8230-8240, 8243-8246, 8249, 8255, 8260-8263, 8310, 8323, 8341, 8380, 8430, 8440, 8460, 8470, 8480-8481, 8490, 8500, 8507, 8510, 8560, 8570, 8574, 8576, and 8936) were used to select patients.

All of the patients included in the study received surgery, but they did not receive any other type of cancer treatment, including radiation therapy, hormone therapy, immunotherapy, palliative care, or any other therapy apart from chemotherapy. All other therapies except for chemotherapy were excluded. Only patients treated exclusively with chemotherapy were included. 

The colon cancer patient's survival duration was calculated using the date of diagnosis to the date of death, date of the failure to follow-up, or the date of the end of the study (December 31, 2020). The variables that were investigated in the study include sex, payer status, race, and age, Charlson comorbidity index (CCI) score, distance traveled income, patient origin, facility type, treatment delay, chemotherapy, stage, and year of diagnosis. 

The ages of the patients were classified separately into two groups: those aged 18 to 59 and those aged ≥60 years. The patient's race was included as either Black or White, and the patient's sex was defined as male or female. The CCI score was categorized as CCI≥1 or CCI=0 to indicate whether a patient did or did not present with a comorbidity at the time of the cancer diagnosis. Patients were classified into different groups based on year 2000 median house income (< $35,000 or ≥ $35,000). Patient insurance was included as private insurance or Medicaid. The distance that was traveled to the treatment facility was grouped as 0 to 19 or ≥ 20 miles. The type of treatment facility was included based on the NCDB’s categorization as either a Comprehensive Community Cancer or an Academic/Research program. Patient origin was defined as being from a metropolitan, urban, or rural area. Treatment delay was defined as the time between diagnosis and treatment and was grouped as either 0 to 29 or ≥30 days. Tumor stages II and III from the NCDB were included. Tumor grade defined from the NCDB data (only available between 2004 and 2017) was included in the study as either moderately differentiated, moderately well differentiated, or intermediately differentiated, or defined as poorly differentiated. The year of diagnosis was grouped as patients who were diagnosed between 2004 and 2011 and between 2012 and 2017. Chemotherapy treatment status was listed as “received” or “not received.” All patients included in this study received surgery but did not receive any radiation, hormonal, transplant, palliative, or any other types of therapy. 

The propensity score was calculated using logistic regression to calculate the probability of patients being in the chemotherapy group. The PSMATCH procedure using the SAS software (SAS Institute, Cary, NC, USA) was used to perform PSM on patients who received chemotherapy to patients who received a treatment other than chemotherapy. The greedy nearest neighbor method of matching matched one chemotherapy patient to one non-chemotherapy patient within a caliper of 0.2. An exact match was done for age, gender, stage, and race at diagnosis. A caliper, such as caliper=0.5, is the required caliper that was used for matching; this means that the difference in PS logits must be ≤0.5 times the estimated logit of the standard deviation. 

Before and after matching, the chi-square test was used to compare patient characteristics between chemotherapy and non-chemotherapy patients. The estimated survival of patients was estimated by using the Kaplan-Meier method. To compare the OS for chemotherapy, the log-rank test was used. At the same time, to estimate the hazard of death for chemotherapy, while adjusting for other factors, multivariate Cox regression was used. Modeling, data management, and statistical analysis were performed using the statistical software SAS 9.4 for Windows (SAS Institute, Cary, NC, USA). All p-values <0.05 or 95% confidence intervals (CI) for the hazard ratio (HR) not including one were considered to be statistically significant. 

## Results

Among the 70,876 patients with stage II and II colon cancer included in this study, 44,992 patients received chemotherapy and 25,884 patients received treatment other than chemotherapy. After PSM, overall, 5,978 patients received chemotherapy, and 5,978 patients did not receive chemotherapy. The patient demographics and clinical characteristics are summarized in Table 1. 

**Table 1:** Patient demographics and clinical characteristics of patients who received chemotherapy and those who did not before and after PSM PSM: Propensity score matching, Mod: Moderately differentiated, Mod Well: Moderately well differentiated, Int: Intermediately differentiated, Poorly: Poorly differentiated Statistically significant: p<0.05

Characteristics	Subtype	Pre-PSM					Post-PSM				
		Non-chemo		Chemo		p-value	Non-chemo		Chemo		p-value
		N	%	N	%		N	%	N	%	
Sex	Male	13416	36.78	23062	63.22	0.4309	5978	50	5978	50	1
	Female	12552	36.49	21846	63.51		5700	50	5700	50	
Race	White	22182	36.75	38183	63.25	0.1539	10020	50	10020	50	1
	Black	3786	36.02	6725	63.98		1658	50	1658	50	
Stage	2	22277	69.19	9922	30.81	<0.0001	8495	50	8495	50	1
	3	3691	9.54	34986	90.46		3183	50	3183	50	
Age (years)	18-59	3656	24.87	11046	75.13	<0.0001	2692	50	2692	50	1
	≥60	22312	39.72	33862	60.28		8986	50	8986	50	
Year of diagnosis	2004-2011	13844	36.63	23949	63.37	0.9688	6459	48.18	6946	51.82	<0.0001
	2012-2019	12124	36.65	20959	63.35		5219	52.45	4732	47.55	
Distance traveled (miles)	0-19	18126	37.04	30807	62.96	0.0094	8638	49.11	8952	50.89	<0.0001
	≥20	5803	35.91	10359	64.09		3040	52.72	2726	47.28	
Facility type	Community	15810	37.08	26829	62.92	0.0028	7483	51.44	7063	48.56	<0.0001
	Academic	10158	35.97	18079	64.03		4195	47.62	4615	52.38	
Treatment delay (days)	0-29	20123	35.25	36962	64.75	<0.0001	8268	45.21	10018	54.79	<0.0001
	≥30	5293	42.37	7200	57.63		3410	67.26	1660	32.74	
Insurance	Private	22986	36.40	40155	63.60	0.0002	9324	47.5	10304	52.5	<0.0001
	Medicaid	2982	38.55	4753	61.45		2354	63.14	1374	36.86	
Income ($1000)	<35	7295	37.76	12026	62.24	0.0007	5265	58.68	3707	41.32	<0.0001
	≥35	16008	36.34	28042	63.66		6413	44.58	7971	55.42	
Patient origin	Urban	21748	36.79	37359	63.21	0.0123	9724	48.92	10153	51.08	<0.0001
	Rural	3394	35.46	6177	64.54		1954	56.17	1525	43.83	
Comorbidity	No	18743	34.61	35411	65.39	<0.0001	8054	47.04	9069	52.96	<0.0001
	Yes	7225	43.21	9497	56.79		3624	58.14	2609	41.86	
Grade	Mod, Mod Well, Int	21897	38.93	34349	61.07	<0.0001	9770	51.79	9094	48.21	<0.0001
	Poorly	4071	27.83	10559	72.17		1908	42.48	2584	57.52	

Before PSM, there was no statistically significant difference found in those receiving chemotherapy based on sex, race, year diagnosed, and grade. For example, between males and females, there was no statistically significant difference in receiving chemotherapy, with 23,062 patients and 21,846 patients, respectively (p>0.05). However, there was a significant difference in receiving chemotherapy found between age (years), treatment delay (days), comorbidity, distance traveled, patient origin, insurance, income, and stage (p<0.05). 

After PSM, the number of patients was reduced from 70,000 patients to about 23,000 patients. With exact matching for sex, race, age, and stage, a statistically significant difference was found when comparing those receiving chemotherapy in all other variables (p<0.0001) listed in Table 2. There was a significant difference in treatment delay between those receiving chemotherapy between 0 and 29 (10,018 patients) and ≥30 days (1,660 patients) (p<0.0001). There was no significant difference in receiving chemotherapy when comparing analytic tumor stage II and III at 8,495 patients each (p=1). After matching, our data demonstrated that the chemotherapy and non-chemotherapy groups had similar patient demographics and stage at diagnosis. 

**Table 2:** Multivariate analysis of predictors of OS prior to and after PSM OS: Overall survival, PSM: Propensity score matching, Mod: Moderately differentiated, Mod Well: Moderately well differentiated, Int: Intermediately differentiated, Poorly: Poorly differentiated Statistically significant: p<0.05

Characteristic	Subtype	Pre-PSM				Post-PSM			
		HR	95% CI		p-value	HR	95% CI		p-value
			Lower	Upper			Lower	Upper	
Sex	Male	1.18	1.15	1.21	<0.0001	1.18	1.13	1.23	<0.0001
	Female	1.00	-	-	-	1	-	-	-
Race	White	1.00	-	-	-	1	-	-	-
	Black	1.05	1.01	1.09	0.0139	1.04	0.97	1.10	0.2864
Age (years)	18-59	1.00	-	-	-	-	-	-	-
	≥60	1.66	1.60	1.73	<0.0001	2.12	1.97	2.63	<0.0001
Year of diagnosis	2004-2011	1.00	-	-	-	-	-	-	-
	2012-2019	0.87	0.85	0.90	<0.0001	0.89	0.85	0.94	<0.0001
Distance traveled (miles)	0-19 miles	1.00	-	-	-	-	-	-	-
	20+ miles	0.99	0.95	1.03	0.5256	0.97	0.92	1.04	0.3938
Facility type	Community	1.00	-	-	-	-	-	-	-
	Academic	0.94	0.92	0.97	<0.0001	0.92	0.88	0.96	0.0003
Treatment delay (days)	0-29	1.00	-	-	-	-	-	-	-
	≥30	0.94	0.90	0.97	0.0005	0.94	0.89	0.99	0.0227
Insurance	Private	1.00	-	-	-	-	-	-	-
	Medicaid	1.53	1.47	1.59	<0.0001	1.52	1.44	1.61	<0.0001
Income	<35k	1.11	1.08	1.15	<0.0001	1.07	1.03	1.13	0.0029
	≥35k	1.00	-	-	-	-	-	-	-
Patient origin	Metro	1.00	-	-	-	-	-	-	-
	Urban/Rural	0.99	0.94	1.04	0.6037	0.92	0.91	1.06	0.6140
Comorbidity	No	1.00	-	-	-	-	-	-	-
	Yes	1.50	1.45	1.54	<0.0001	1.51	1.44	1.58	<0.0001
Grade	Mod, Mod Well, Int	1.00	-	-	-	-	-	-	-
	Poorly	1.43	1.39	1.48	<0.0001	1.40	1.334	1.48	<0.0001
Stage	2	1.00	-	-	-	-	-	-	-
	3	2.09	2.01	2.17	<0.0001	2.17	2.08	2.27	<0.0001
Chemo	No	1.00	-	-	-	-	-	-	-
	Yes	0.51	0.49	0.53	<0.0001	0.54	0.52	0.57	<0.0001

**Figure 1:**
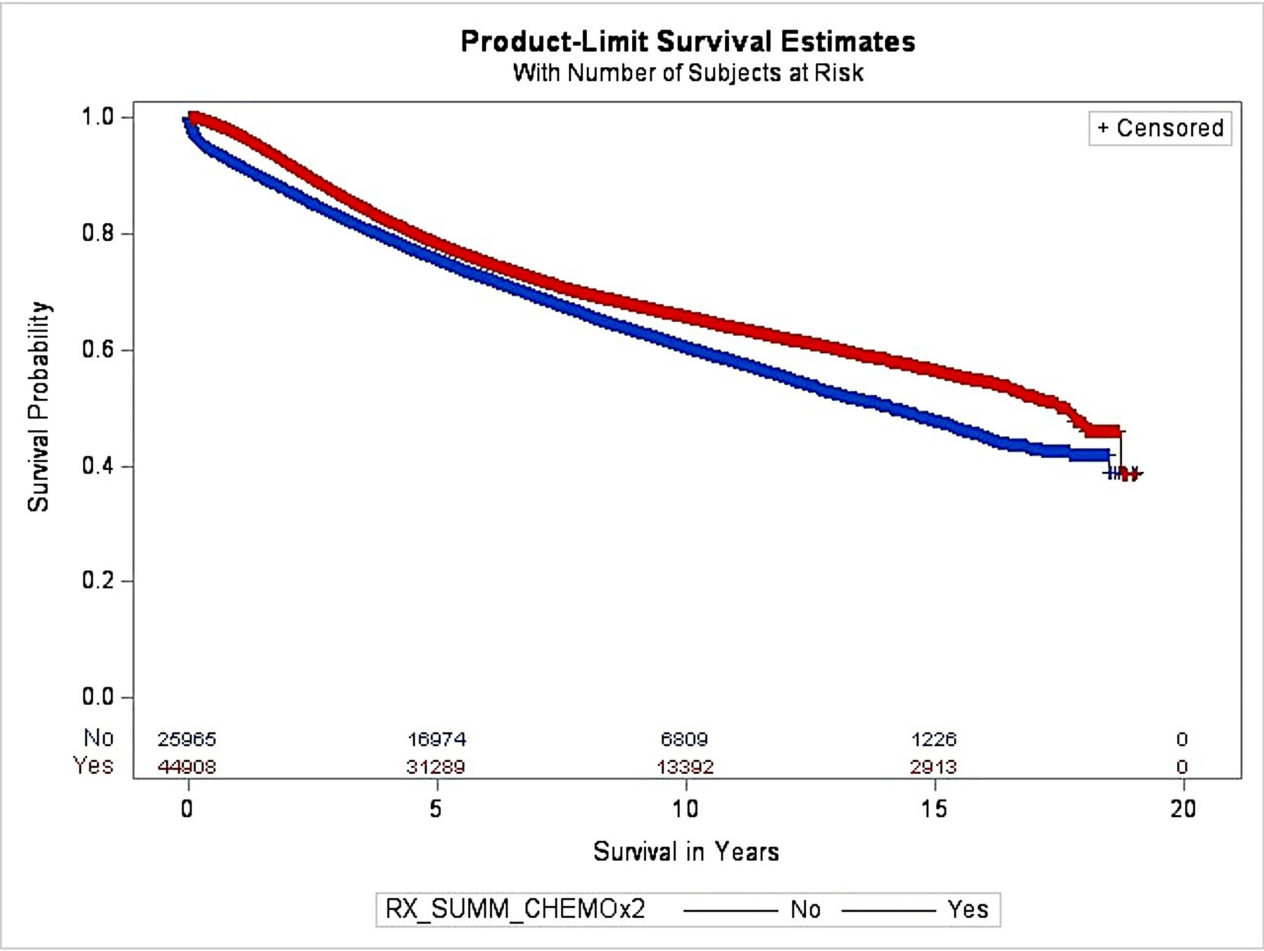
The OS before PSM for the chemotherapy group OS: Overall survival, PSM: Propensity score matching

The univariate analysis of OS according to chemotherapy before and after PSM is presented in Figure 1. It shows that before PSM, the median overall survival (MOS) among chemotherapy and non-chemotherapy groups was 17.55 years and 14.12 years, respectively. Before the PSM method was used, multivariate COX regression analysis was also used, adjusting for other factors, the results of which are listed above in Table 2. Chemotherapy was a significant predictor of patients’ survival outcomes. The HR was 0.51 for patients receiving chemotherapy compared to patients who did not receive chemotherapy. It demonstrated that patients receiving chemotherapy were 49% less likely to die as compared to patients without chemotherapy. 

**Figure 2:**
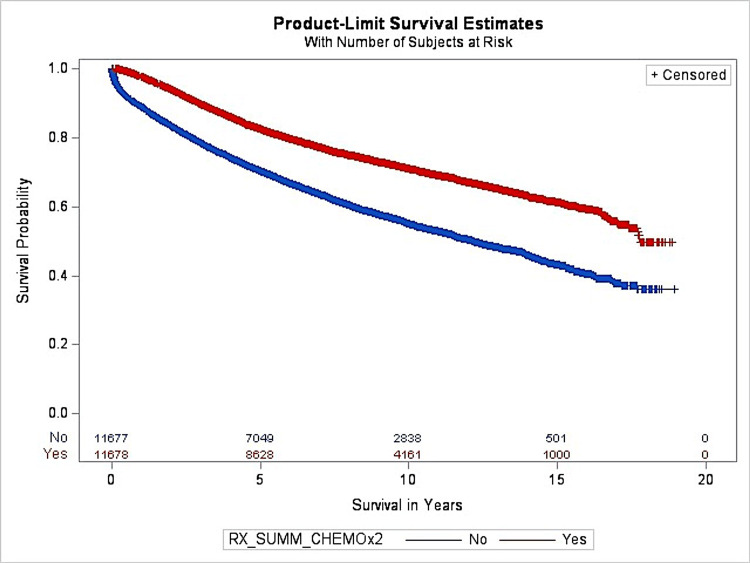
The OS after PSM for the chemotherapy group OS: Overall survival, PSM: Propensity score matching

Figure 2 shows the OS after PSM for patients who underwent chemotherapy. The MOS was 17.77 years for the chemotherapy group and 12.18 years for the non-chemotherapy group. After matching, chemotherapy was determined to play an integral role in the OS of colon cancer patients. 

After PSM, the HR was 0.54 for patients who received chemotherapy compared to patients who did not receive chemotherapy. Similar results were discovered after adjusting for factors including age, race, gender, distance traveled, year of diagnosis, stage, grade, comorbidity, treatment delay, income, facility type, patient origin, and insurance. After PSM, factors such as sex, age, year, insurance, comorbidity, ATS, and chemotherapy were found to be significant predictors in OS for patients with colon cancer. 

## Discussion

Of the 70,876 patients, 44,992 patients in our study underwent chemotherapy. Specifically for tumor stages II and III colon cancers included in the study, our data showed that the usage of chemotherapy before and after PSM was 9,922 and 34,986 patients, respectively. Before PSM, using multivariate Cox regression analysis, it was indicated that patients who received chemotherapy were 49% less likely to die when compared to those who did not receive chemotherapy. After PSM, multivariate Cox regression analysis showed that those who received chemotherapy were 46% less likely to die, which is similar to the results found in a study by Yan et al [21]. Our results determined that chemotherapy truly was a significant predictor of OS in patients with colon cancer. 

The results of Table 1 demonstrate that, before PSM, other than sex, race, and year of diagnosis, there was a statistically significant difference found between all other variables included in this study in receiving chemotherapy. A difference in receiving chemotherapy among variables such as stage, age, distance traveled, facility type, treatment delay, insurance, comorbidity, and grade was consistent with the results found in other studies [19,22-25]. The results of Figure 1 show that before PSM, there was a statistically significant difference found in OS between chemotherapy and non-chemotherapy groups, which was similar to the results found in a study by Liu et al. [26]. 

After PSM, the results in Table 1 showed that besides age, race, stage, and sex, which were exact matches, a statistically significant difference was found between receiving chemotherapy and all other variables. For the exact match variables, there is no association between the variables and patients receiving chemotherapy. In this case, the baseline characteristics between patients receiving chemotherapy and patients not receiving chemotherapy are similar in these four variables. If the OS was found to be associated with chemotherapy, it means that the OS was not due to variation in the variables that were matched. It indicates that the variable itself has a direct impact on OS. For example, for tumor stage, there was no significant difference in receiving chemotherapy between stage II and III patients after PSM. Nonetheless, our data indicates that patients with stage III cancer at the time of diagnosis were 2.17 times more likely to die compared to patients with stage II cancer, which is similar to the results found by Maringe et al. [8]. The HR increased from 2.09 to 2.17 before and after PSM, respectively. Similarly, after PSM, patients aged greater than 60 were 2.12 times as likely to die when compared to patients aged between 18 and 59, and this was consistent with other studies [25,27,28]. As compared to age <60, for patients aged >60, the HR increased from 1.66 before PSM to 2.12 after PSM. Furthermore, after PSM, our data showed that patients with Medicaid and patients presenting with comorbidities at the time of diagnosis were significant predictors for worse OS, which is similar to the data found in other studies [19,28,29]. Our study shows differences in the HR before and after PSM, which we interpreted as becoming a more accurate estimation of the risk of dying among many investigated factors. 

Even though the PSM method was used in assessing the effects of chemotherapy on colon cancer patients, the matching was performed on four factors that the authors believe to be important. The study could be strengthened if more factors associated with colon cancer survival could be studied. As a hospital-based study, it did present some limitations. First, the NCDB only includes about 70% of new patients diagnosed with cancer in this country, which means that we do not have the data on the other 30% of patients diagnosed with cancer. Second, there were over 200 variables collected for the 2004-2021 data set, we only investigated 14 variables that we considered to be appropriate for the aims of our study. This is partly because some variables in the NCDB have only been collected in recent years. Understandably, including more variables in the study may more accurately estimate the effect of chemotherapy on OS. Third, some of the variables have missing data values, which limited the number of cases included in our study. Fourth, certain variables, like income, were based on zip code instead of individual or household income and may not have accurately reflected the individual patient’s income. Fifth, our study listed the chemotherapy variable for those who did receive chemotherapy simply as 'yes,' while the chemotherapy group in the NCDB further includes single agent, double agent,t or triple agent. Further, we do not have the exact name of the agents used for chemotherapy in the NCDB, so the chemotherapy agents may have changed over time. Future prospective clinical trials may be needed to more accurately estimate the effects of chemotherapy on OS in patients with stage II and II colon cancer. 

## Conclusions

The results of this study indicate that after PSM, patient age, sex, race, and stage were similar between those who did and did not receive chemotherapy. With these similarities in baseline characteristics between chemotherapy and non-chemotherapy groups after PSM, the HR demonstrated that patients receiving chemotherapy were 46% less likely to die. We believe that our results show that receiving chemotherapy is a significant predictor of OS in patients with colon cancer after adjusting for other factors. 
